# Early onset of efficacy with erenumab in patients with episodic and chronic migraine

**DOI:** 10.1186/s10194-018-0923-6

**Published:** 2018-10-01

**Authors:** Todd Schwedt, Uwe Reuter, Stewart Tepper, Messoud Ashina, David Kudrow, Gregor Broessner, Guy P. Boudreau, Peter McAllister, Thuy Vu, Feng Zhang, Sunfa Cheng, Hernan Picard, Shihua Wen, Joseph Kahn, Jan Klatt, Daniel Mikol

**Affiliations:** 1 Department of Neurology, Mayo Clinic, 5777 E Mayo Blvd, Phoenix, AZ 85054 USA; 2 Department of Neurology, Charité Universitätsmedizin Berlin, Berlin, Germany; 3 Geisel School of Medicine at Dartmouth, Hanover, NH USA; 4 Danish Headache Center and Department of Neurology, Rigshospitalet Glostrup, Faculty of Medical and Health Sciences, University of Copenhagen, Copenhagen, Denmark; 5 California Medical Clinic for Headache, Santa Monica, CA USA; 6 Department of Neurology, Headache Outpatient Clinic, Medical University of Innsbruck, Innsbruck, Austria; 7 Clinique de la Migraine et Céphalées, Département de Neurologie, Centre Hospitalier de L’Université de Montréal, Hôpital Notre-Dame, Montréal, QC Canada; 8 New England Institute for Neurology & Headache, Stamford, CT USA; 9 Amgen Inc., Thousand Oaks, CA USA; 10 Novartis Pharmaceuticals Corp., East Hanover, NJ USA; 11 Novartis Pharma AG, Basel, Switzerland

**Keywords:** Erenumab, Chronic migraine, Episodic migraine, Efficacy, Migraine preventive medication, Onset of efficacy, Migraine preventive clinical trial

## Abstract

**Background:**

Subcutaneous erenumab reduced monthly migraine days and increased the likelihood of achieving a ≥ 50% reduction at all monthly assessment points tested in 2 pivotal trials in episodic migraine (EM) and chronic migraine (CM). Early efficacy of migraine preventive medications is an important treatment characteristic to patients. Delays in achievement of efficacy can result in failed adherence. The objective of these post-hoc analyses were to evaluate efficacy in the first 4 weeks after initial subcutaneous administration of erenumab 70 mg, erenumab 140 mg, or placebo.

**Methods:**

There is no generally accepted methodology to measure onset of action for migraine preventive medications. We used a comprehensive approach with data from both studies to evaluate change from baseline in weekly migraine days (WMD), achievement of ≥ 50% reduction in WMD, and proportion of patients experiencing migraine measured on a daily basis. The 7-day moving averages were overlaid with observed data.

**Results:**

In both studies (EM: *N* = 955; CM: *N* = 667), there was evidence of onset of efficacy of erenumab vs. placebo during the first week of treatment, which in some cases reached nominal significance. For EM the changes in WMD were (least squares mean [LSM] [95% CI]): placebo, − 0.1 (− 0.3, 0.0); erenumab 70 mg, − 0.3 (− 0.5, − 0.2) *p* = 0.130; erenumab 140 mg, − 0.6 (− 0.7, − 0.4) *p* < 0.001. For CM the changes were: placebo, − 0.5 (− 0.8, − 0.3); erenumab 70 mg, − 0.9 (− 1.2, − 0.7) *p* = 0.047; erenumab 140 mg, − 0.8 (− 1.1, − 0.5) *p* = 0.18. Achievement of ≥ 50% reduction in WMD was observed as early as Week 1 (adjusted OR [95% CI] erenumab vs placebo) in EM: erenumab 70 mg, 1.3 (1.0, 1.9) *p* = 0.097; erenumab 140 mg, 2.0 (1.4, 2.7) *p* < 0.001. A similar outcome was observed for CM: erenumab 70 mg, 1.8 (1.1, 2.8) *p* = 0.011; erenumab 140 mg, 1.9 (1.2, 2.9) *p* = 0.009. Seven-day moving averages of observed data showed each treatment arm differed from placebo by Week 1 (OR [95% CI]): in EM Day 3 for erenumab 140 mg, 0.7 (0.5, 1.0) *p* = 0.031 and at Day 7 for 70 mg, 0.6 (0.4, 0.8) *p* = 0.002; in CM: Day 6 for erenumab 70 mg, 0.6 (0.4, 0.9) *p* = 0.022 and at Day 7 for 140 mg, 0.7 (0.4, 1.0); *p* = 0.038.

**Conclusion:**

Erenumab showed early onset of efficacy with separation from placebo within the first week of treatment in both chronic and episodic migraine patients.

## Background

Migraine can result in severe disability with substantial burden for patients and families [1, 2]. Disruptions to work life, activities of daily living, social and leisure activities, and physical and emotional functioning may occur both during and between migraine attacks [3, 4]. Migraine-related disability and burden is present in patients with episodic migraine (EM) and those with chronic migraine (CM), and studies show patients prefer effective and well-tolerated preventive treatments with rapid onset of action [5].

In order to avoid adverse effects, most commonly prescribed preventive medications (e.g. beta-blockers, tricyclic antidepressants, topiramate, and valproate) require titration, and once the proper dose is attained, efficacy can still be delayed. This delay in efficacy, coupled with tolerability issues, contributes to poor adherence and, ultimately, failed migraine prevention. Achieving rapid efficacy of c migraine preventive therapy could reduce the need for acute treatments or even, in some particularly severe situations, transitional therapies (e.g. corticosteroids) that are required when patients have to wait for a migraine preventive therapy to have an effect [6].

Research on migraine neurobiology conducted over the last two decades demonstrated that calcitonin gene-related peptide (CGRP) plays an important role in migraine pathophysiology, and that targeting this pathway can be an effective preventive treatment strategy for migraine [7–9]. Erenumab is a fully human monoclonal antibody (mAb) that binds and inhibits the canonical CGRP receptor [10]. The efficacy and safety of erenumab 70 mg and 140 mg monthly have been shown in a 24-week, double-blind, placebo-controlled clinical trial in EM [11] (STRIVE) and a pivotal 12-week double-blind, placebo-controlled clinical trial in CM [12]. In these 2 studies, both doses of erenumab were effective in reducing monthly migraine days (MMD; primary efficacy measure in both studies) at all monthly time points tested in patients receiving regular treatment every 4 weeks, including the earliest pre-specified time point of Week 4, which suggests that both doses of erenumab could also be effective at even earlier time points [11, 12].

There are no standard methodological approaches for assessing time to initial onset of efficacy in preventive therapies for migraine. Clinical trial endpoints typically compare mean MMD during treatment to MMD during a 1-month baseline. As the earliest pre-specified efficacy time point in erenumab prevention trials was Month 1 (Week 4), in order to further refine our understanding of the time to onset of efficacy of erenumab, we conducted post hoc analyses of these two pivotal studies in EM and CM, during the double blind phase, at time points earlier than Week 4. We focused on migraine day frequency here, as it is the most relevant measure, and it is related to primary and key secondary endpoints in our studies.

## Methods

### The EM and CM studies

**Fig. 1:**
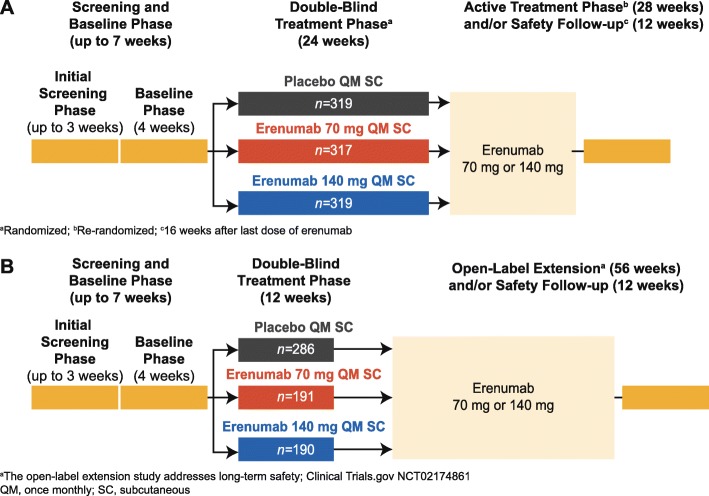
Study design of the episodic migraine trial (**a**) and the chronic migraine trial (**b**)

The EM study (NCT02456740, STRIVE) was an international, randomized, double-blind, placebo-controlled, parallel-group, phase 3 pivotal regulatory trial of erenumab 70 mg and 140 mg administered monthly by subcutaneous (SC) injection in adult patients with EM where randomization was stratified by region and by preventive migraine medication use (Fig. 1a) [11]. The CM study (NCT02066415) was an international, randomized, double-blind, placebo-controlled, parallel-group pivotal regulatory trial in adult patients with CM where randomization was stratified by region and medication overuse (Fig. 1b) [12]. Detailed information on study designs, populations, and results are provided in the primary publications [11, 12]. The two studies were selected for post-hoc analyses on onset of efficacy given their design (i.e. placebo-controlled), sample size and the use of both doses of erenumab, 70 mg and 140 mg, across the two migraine categories, EM and CM.

Each day during the studies, patients completed an electronic diary with information about their migraine and non-migraine headaches, patient reported outcomes (e.g. physical impairment) and use of acute migraine therapies during the 1-month baseline phase and subsequent double-blind treatment phase. The primary endpoint was the change from baseline in MMD averaged over Months 4, 5, and 6 for the EM trial and at Month 3 in the CM trial, with additional pre-specified analyses at the end of each monthly treatment period. The migraine day definition in both studies followed International Classification of Headache Disorders 3rd Edition (ICHD-3) definitions for migraine and probable migraine [13]. The protocol and patient consent information were approved by all relevant ethical review boards, all subjects gave written informed consent, and the studies were conducted in accordance with the principles of the Declaration of Helsinki and Good Clinical Practice [14].

### Statistical analyses

The full methods for statistical analyses of each study were published previously [11, 12]. To assess the efficacy of erenumab versus placebo earlier than Week 4, post hoc analyses evaluated the change from baseline in the number of migraine days per week (WMD) and achievement of ≥ 50% reduction from baseline in WMD in the total study populations.

The baseline mean WMD was calculated on the basis of the entire 4-week baseline period (normalized into a 7-day period). For change from baseline in WMD, the least squares mean (LSM) at each time point was calculated with adjusted analyses using a generalized linear mixed-effects model that included treatment group, visit (week), treatment by visit interaction, stratification factors and average WMD at baseline as covariates. For achievement of ≥ 50% reduction in WMD, odds ratios (ORs) at each weekly time point were estimated using the stratified Cochran-Mantel-Haenszel test with missing data imputed as nonresponse.

To observe trends in efficacy at time points even earlier than the first scheduled visit at Week 4, the daily incidence of migraine during the first 2 weeks was analyzed. The proportion of patients with presence of migraine was plotted for each day during Days 1–14 (first 2 weeks of the double-blind treatment phases of the studies). The slopes and moving averages (7-day average, taking into account observations 3 days before and after a given day) were calculated and overlaid with observed data. In addition, the ORs of migraine presence for each day were estimated for the first 2 weeks using a generalized linear mixed-effects model that included treatment group, visit (day), treatment by visit interaction, stratification factors and baseline values (presence of migraine on the day prior to the first dose) as covariates.

*P*-values for these post-hoc endpoints are based on ORs or LSM differences from placebo and are not adjusted for multiple comparisons. Statistical significance was determined by comparing descriptive *p*-values with nominal significance level at *p* ≤ 0.05.

## Results

### Change from baseline in weekly migraine days

**Fig. 2:**
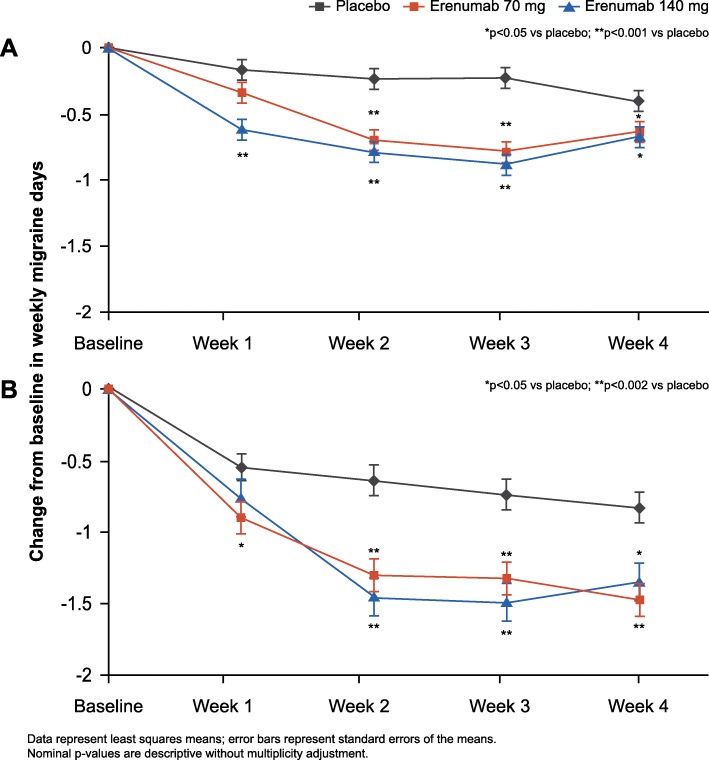
Change from baseline in WMD during the first month of studies in episodic migraine (**a**) and chronic migraine (**b**)

The WMD at baseline were 2.1 ± 0.6 for EM, and 4.5 ± 1.2 for CM. In the EM study, erenumab treatment was associated with nominally significant reductions in WMD compared to placebo as early as Week 1 and for all doses by Week 4; (LSM change from baseline [95% CI]) in the EM study: placebo (− 0.1 [− 0.3, 0.0]), 70 mg (− 0.3 [− 0.5, − 0.2]; *p* = 0.130) and 140 mg (− 0.6[− 0.7, − 0.4]; *p* < 0.001) for Week 1; placebo (− 0.4 [− 0.5, − 0.2]), 70 mg (− 0.6 [− 0.8, − 0.5]; *p* = 0.029) and 140 mg (− 0.6 [− 0.8, − 0.5]; *p* = 0.019) at Week 4. For CM, erenumab treatment was associated with nominally significant reductions in WMD compared to placebo for both doses at Week 1 and efficacy was sustained through Week 4; (LSM change from baseline [95% CI]) in the CM study: placebo (− 0.5 [− 0.8, − 0.3]), 70 mg (− 0.9 [− 1.2, − 0.7]; *p* = 0.047) and 140 mg (− 0.8 [− 1.1,− 0.5]; *p* = 0.18) for Week 1; placebo (− 0.8 [− 1.0, − 0.6]), 70 mg (− 1.5 [− 1.7, − 1.2]; *p* < 0.001) and 140 mg (− 1.4 [− 1.6, − 1.1]; *p* = 0.002) at Week 4 (Fig. 2).

### ≥ 50% reduction from baseline in weekly migraine days

**Fig. 3:**
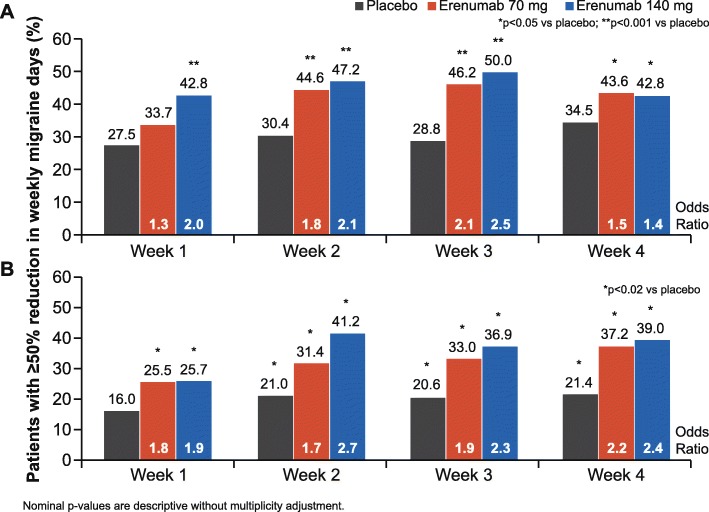
Proportion of patients with ≥ 50% reduction from baseline in WMD during the first month of studies in episodic migraine (**a**) and chronic migraine (**b**)

In both studies, the odds of achieving a ≥ 50% reduction from baseline in WMD were higher in patients who received erenumab compared with those who received placebo as early as the first week and sustained through Week 4 (adjusted OR [95% CI]) for erenumab versus placebo in the EM study: 70 mg (1.3 [1.0, 1.9]; *p* = 0.097) and 140 mg (2.0 [1.4, 2.7]; *p* < 0.001) for Week 1, 70 mg (1.5 [1.1, 2.0]; *p* = 0.020) and 140 mg (1.4 [1.0, 2.0]; *p* = 0.033) at Week 4; adjusted OR (95% CI) vs placebo in the CM study: 70 mg (1.8 [1.1, 2, 8]; *p* = 0.011) and 140 mg (1.9 [1.2, 2.9]; *p* = 0.009) for Week 1, 70 mg (2.2 [1.5, 3.3]; *p* < 0.001) and 140 mg (2.4 [1.6, 3.5]; *p* < 0.001) at Week 4 (Fig. 3).

### Proportion of patients with a migraine day and 7-day moving averages

**Fig. 4:**
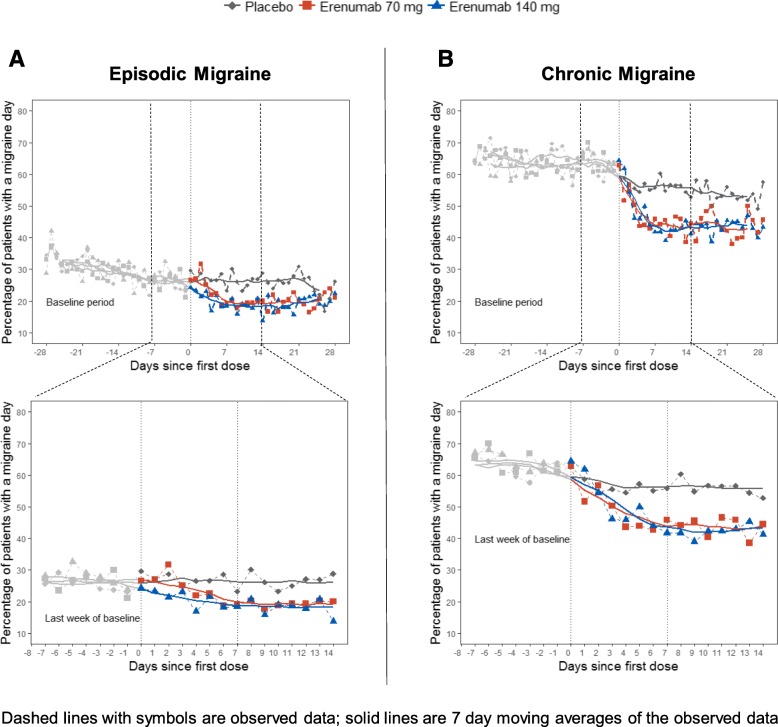
Percentage of patients with episodic migraine (**a**) chronic migraine (**b**) with a migraine day and 7-day moving averages for the 4-week baseline period and first 4-weeks of double-blind treatment (top panel) and for the last week of baseline and first 2 weeks of double-blind treatment (bottom panel)

Although there was some variability between the studies regarding the day of efficacy onset for erenumab, efficacy was observed within the first week following treatment initiation in both studies and was sustained through Week 4 (Fig. 4). In the EM study, the first occurrence of significance comparing the odds of having a migraine day versus placebo was at Day 3 for erenumab 140 mg (OR [95% confidence interval (CI)]: 0.67 [0.47, 0.96]; *p* = 0.031) and at Day 7 for 70 mg (OR [95% CI]: 0.55 [0.38, 0.80]; *p* = 0.002). In the CM study, the first occurrence of significance comparing the odds of having a migraine day versus placebo was at Day 6 for erenumab 70 mg (OR [95% CI]: 0.62 [0.42, 0.93]; *p* = 0.022) and at Day 7 for 140 mg (OR [95% CI]: 0.65 [0.43, 0.98]; *p* = 0.038). Moreover, 7-day moving averages of observed data of each erenumab treatment arm were separated from placebo within the first week (Fig. 4).

## Discussion

In this manuscript we report post hoc results of three different sets of analyses from two (EM and CM) studies: WMD, ≥ 50% reduction in WMD, and proportion of subjects with a migraine on a given day (time to the first day with nominally significant reduction in odds for having a migraine for erenumab compared to placebo). The totality of these exploratory analyses supports a time to onset of efficacy of erenumab within the first week in both EM and CM. These effects were sustained through Week 4, prior to the next monthly dose of erenumab, and continued throughout the double-blind phases of both studies at all time points [11, 12] . Additionally, at Week 1, 43% (EM) and 26% (CM) of patients in the erenumab 140 mg group experienced a ≥ 50% reduction in WMD (15% increase vs placebo [EM] and 10% increase vs placebo [CM]) (Fig. 3).

Since there is no commonly accepted methodology for measuring onset of efficacy in migraine prevention, several analytical approaches were used in this study. These analyses support the onset of the efficacy of erenumab versus placebo in the week after the first injection. For weekly analyses of change in migraine frequency and ≥ 50% response rates, point estimates were slightly lower for Week 1 than for Week 2; moreover, the efficacy estimates at the end of Week 1 were attenuated by the inclusion of results for the first 2–3 days (when plasma concentrations of erenumab were still rising). Figure 4 shows that efficacy at the end of Week 1 was greater than efficacy at the beginning of Week 1 and comparable to efficacy observed in Week 2.

The data presented here are consistent with the pharmacokinetic profile of erenumab following SC injection [15]. A population pharmacokinetic model (based on data from EM and CM studies) was used to fit observed data to full pharmacokinetic profiles. For SC dosing, the absorption half-life was estimated at 2.3 days, yielding time to maximal concentration of 4–11 days (with higher maximal serum concentration [C_max_] for 140 mg). Considering the median pharmacokinetic profile, systemic erenumab concentrations already exceed relevant clinical levels (i.e. Week 12 C_min_ under a 70 mg monthly regimen) by Day 2 following a single 140 mg SC dose, or by Day 3 following a 70 mg SC dose.

Since the efficacy of migraine preventive medications within the first few weeks of initiation has typically not been analyzed and/or reported, it is difficult to compare the results of the analyses reported herein to existing migraine medications; however, some data are available for fremanezumab, eptinezumab, and galcanezumab. Fremanezumab (Teva Pharmaceuticals) has shown onset of efficacy results in post hoc analyses from a study of 261 patients with CM [16]. In this study, a different analytical approach was used based on headache hours. There was a significant decrease in the number of headache hours starting 3 days after the highest dose (900 mg) was given (a dose not used in phase 3 trials), and 7 days after the lower dose (675/225 mg) was given. For moderate or severe headache days, a significant decrease was seen during the second week for both doses.

Initial onset of efficacy studies with eptinezumab (Alder BioPharmaceuticals Inc) have been reported as an abstract at a scientific meeting [17]. The study evaluated the effects of eptinezumab or placebo on the likelihood of having a migraine on any given day over each of the first 4 weeks in 1072 subjects with CM. On day 1 post intravenous infusion, the proportions of individuals having a migraine were lower in those receiving eptinezumab 100 mg (28.6%) or eptinezumab 300 mg (27.8%) compared to those receiving placebo (42.3%) [17].

Data on the initial onset of efficacy with galcanezumab (Eli Lilly) were presented in abstract form at a scientific meeting [18]. In this post hoc analysis of data from a phase 2a study of individuals with EM, migraine headache days per week were reduced more in those receiving LY2951742 (*n* = 106) compared to placebo (*n* = 110) as early as Week one.

One strength of the analyses presented in this study is that several complementary statistical approaches were used to explore the onset of efficacy of erenumab from the first days of treatment in both EM and CM patients. A comprehensive approach including the entire study population was used. A limitation of this study is its post hoc nature. Although the trials were powered for the primary endpoint and were controlled for multiplicity for all secondary endpoints, they were not powered for the type of analyses presented here. Further, post hoc analyses are typically not adjusted for multiple comparisons as they are exploratory, rather than confirmatory in nature [19–21]. In this context, presented *p*-values are nominal and should be considered cautiously.

## Conclusions

In patients with either EM or CM, treatment with erenumab showed onset of efficacy within the first week after the first dose, based on a series of complementary analytical approaches and (clinically) relevant efficacy measures (i.e. WMD and ≥ 50% reduction of WMD). After the first week, efficacy was sustained and separation from placebo continued thereafter for the rest of the study as reported separately [11, 12]. In addition to the favourable benefit-risk profile of erenumab, its early onset of efficacy may prove an important benefit to patients with a high burden of disease.

## Abbreviations

CGRP: Calcitonin gene-related peptide
CI: Confidence interval
CM: Chronic migraine
EM: Episodic migraine
ICHD-3: International Classification of Headache Disorders 3rd edition
LSM: Least squares mean
MMD: Monthly migraine days
OR: Odds ratio
SC: Subcutaneous
WMD: Weekly migraine days
